# Comparison of blood‐based biomarkers of AD in individuals following plant‐based diets compared to regular meat eaters

**DOI:** 10.1002/alz70856_103690

**Published:** 2025-12-26

**Authors:** Shaun Eslick, Grace Austin, Jessica Ferguson, Christopher Oldmeadow, Manohar Garg, Ralph N Martins

**Affiliations:** ^1^ Macquarie University, Macquarie Park, NSW, Australia; ^2^ University of Newcastle, Callaghan, NSW, Australia; ^3^ Hunter Medical Research Institute, New Lambton, NSW, Australia; ^4^ Macquarie University, Sydney, NSW, Australia; ^5^ Edith Cowan University, Perth, Western Australia, Australia

## Abstract

**Background:**

Research indicates that plant‐based diets (PBDs) may be protective against neurodegenerative disease such as Alzheimer's disease. Subsequently, this study examined associations between blood‐based AD biomarkers in individuals habitually following different PBDs compared to regular meat‐eating diets (RMEs).

**Method:**

This study is a secondary analysis of the Plant‐based Diets study. Aβ1‐42/Aβ1‐40, *p*‐tau181, NFL and GFAP were measured in 237 plasma samples via Ultra‐sensitive Single Molecule array (SIMOA) from individuals who habitually followed vegan, pesco‐vegetarian (PVs), lacto‐ovo vegetarian (LOVs), semi‐vegetarian (SVs), or regular meat eater diets. Multivariable regression analyses were undertaken to adjust for age and sex.

**Result:**

Plasma Aβ1‐42/Aβ1‐40 ratio was significantly higher in PVs 0.011 (CI: 0.006, 0.016, *p* < 0.01), LOVs 0.011 (CI: 0.007, 0.016, *p* < 0.01) and SVs 0.015 (0.009‐0.020, *p* < 0.01) groups compared to RMEs, following adjustment for age and sex. Plasma *p*‐tau181(pg/mL) was significantly higher in PVs 3.400 (CI: 0.427‐6.364, *p* < 0.05) and LOVs 7.140 (CI: 0.007, 0.016, *p* < 0.01), NFL (pg/mL) higher in PVs 5.177 (CI: 1.645, 8.710, *p* < 0.01) and LOVs 4.013 (CI: 1.554, 6.473, *p* =  0.01), and GFAP (pg/mL) higher in PVs 26.362 (CI: 5.638, 47.086, *p* < 0.05) and LOVs 20.796 (4.950, 36.643, *p* =  0.01), all compared to RMEs.

**Conclusion:**

Findings suggest that PBDs may be associated with blood‐based AD biomarkers, independent of age of sex. Higher plasma levels of Aβ1‐42/Aβ1‐40 in PBDs compared to RMEs, indicated that PBDs might be associated with lesser amyloid burden. However, higher levels of other plasma AD biomarkers were present in some PBDs compared to RMEs. Future studies should aim to examine associations between specific nutrients and biomarkers of AD to further elucidate the role of diet quality and patterns in AD pathology.

